# Role of Sirtuin 6 in the Pathogenesis of Metabolic Dysfunction-Associated Steatotic Liver Disease

**DOI:** 10.3390/cimb48050435

**Published:** 2026-04-22

**Authors:** Yeonsoo Kim, Seonghwan Hwang

**Affiliations:** College of Pharmacy and Research Institute for Drug Development, Pusan National University, Busan 46241, Republic of Korea; sum270@pusan.ac.kr

**Keywords:** epigenetics, histone deacetylation, hepatic lipogenesis, fatty acid oxidation, inflammation, fibrosis

## Abstract

Metabolic dysfunction-associated steatotic liver disease (MASLD) is a leading cause of chronic liver disease worldwide and arises from systemic metabolic dysregulation and insulin resistance. Despite its increasing prevalence, effective pharmacological interventions remain limited. Recent evidence has identified sirtuin 6 (SIRT6), an NAD^+^-dependent epigenetic regulator, as an important modulator of hepatic metabolic and stress-responsive pathways. This review summarizes current knowledge regarding the role of SIRT6 in liver physiology and MASLD pathogenesis. Accumulating evidence indicates that SIRT6 suppresses lipogenic transcriptional programs while enhancing mitochondrial oxidative capacity and fatty acid oxidation, thereby maintaining metabolic homeostasis. Beyond lipid metabolism, SIRT6 is implicated in the regulation of endoplasmic reticulum stress responses, inflammatory signaling, and chromatin accessibility, which are the processes that collectively influence hepatocellular injury and disease progression. In addition, emerging data suggest that SIRT6 modulates hepatic stellate cell activation and fibrogenic signaling pathways, thereby linking epigenetic regulation to the development of liver fibrosis. A reduction in hepatic SIRT6 expression and activity has been reported in metabolic disorders, including MASLD. We further discuss the therapeutic potential of targeting SIRT6, including the development of selective small-molecule activators and naturally derived compounds aimed at restoring SIRT6 activity. Together, the available evidence positions SIRT6 as an important regulatory node in MASLD and a promising candidate for future therapeutic intervention.

## 1. Introduction

Metabolic dysfunction-associated steatotic liver disease (MASLD) is the most prevalent chronic liver disease worldwide and closely parallels the global increase in metabolic syndromes, including obesity, type 2 diabetes mellitus (T2DM), and cardiometabolic disorders [1]. MASLD encompasses a disease spectrum ranging from simple hepatic steatosis to metabolic dysfunction-associated steatohepatitis (MASH), fibrosis, cirrhosis, and hepatocellular carcinoma [2]. Liver-related morbidity and mortality attributable to MASLD continue to rise, establishing MASLD as a major and expanding public health challenge.

MASLD represents the hepatic manifestation of systemic metabolic dysfunction and is closely associated with insulin resistance, dyslipidemia, chronic low-grade inflammation, and impaired energy homeostasis [3]. Although the updated disease nomenclature emphasizes the metabolic basis of the condition [4], therapeutic development has not progressed in parallel with advances in mechanistic understanding. Currently approved and late-stage pharmacological agents primarily target individual downstream pathways, such as thyroid hormone receptor signaling, incretin-based mechanisms, and hepatic lipid flux [5]. As a result, these approaches often address individual disease features rather than the integrated molecular networks that govern disease initiation, progression, and interindividual heterogeneity.

The pathogenesis of MASLD is now recognized as a multifactorial process driven by the interplay of multiple converging mechanisms, including hepatic insulin resistance, lipotoxicity, mitochondrial dysfunction, oxidative stress, endoplasmic reticulum (ER) stress, altered gut–liver signaling, and dysregulated immune activation [6]. These processes operate within a permissive genetic and epigenetic framework that influences disease susceptibility and trajectory. Epigenetic regulators are particularly relevant in this context, as they integrate nutrient availability and metabolic stress with transcriptional programs that coordinate lipid metabolism, inflammatory responses, and cellular stress adaptation [7].

Among these regulators, sirtuin 6 (SIRT6) has emerged as an important metabolic and epigenetic integrator linking chromatin regulation, hepatic lipid metabolism, mitochondrial function, inflammatory signaling, and genome stability [8]. SIRT6 activity is tightly coupled to cellular nicotinamide adenine dinucleotide (NAD^+^) availability, positioning it as both a sensor and effector of metabolic state [8,9]. Accumulating experimental and clinical evidence indicates that hepatic SIRT6 expression and activity are reduced in metabolic disease states, including MASLD, and that loss of SIRT6 function exacerbates steatosis, inflammation, fibrogenesis, and hepatocellular carcinogenesis [10,11,12].

In this review, we summarize current concepts of MASLD pathogenesis and examine the mechanistic roles of SIRT6 in hepatic metabolic regulation and disease progression. We further discuss emerging therapeutic strategies aimed at restoring or enhancing SIRT6 activity, highlighting its potential as a disease-modifying target capable of addressing the interconnected metabolic, inflammatory, and fibrogenic pathways underlying MASLD.

## 2. MASLD Pathogenesis

**Figure 1:**
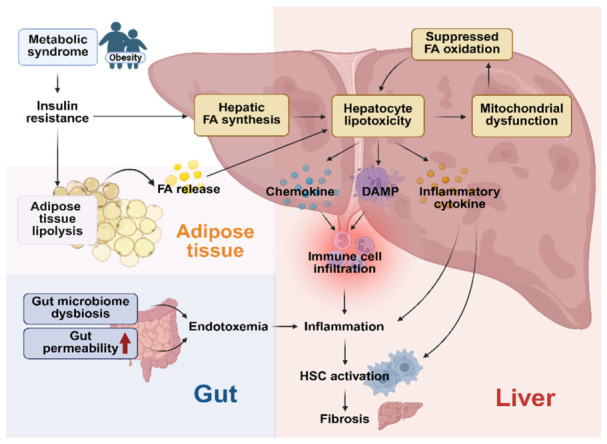
Multi-organ mechanisms underlying the pathogenesis of MASLD. Excess nutrient intake and metabolic dysfunction promote adipose tissue lipolysis, leading to increased free fatty acid (FA) flux to the liver and enhanced hepatic lipid synthesis. In parallel, gut microbiota dysbiosis and increased intestinal permeability contribute to endotoxemia. These factors induce hepatocyte lipotoxicity, mitochondrial dysfunction, and inflammatory signaling, resulting in immune cell infiltration, hepatic stellate cell (HSC) activation, and progression to fibrosis. Created in BioRender. Hwang, S. (2026). BioRender.com/lyf5mku (accessed on 7 March 2026).

MASLD emerges from systemic metabolic dysregulation and represents the hepatic manifestation of whole-body energy imbalance. It is defined by excessive lipid accumulation in hepatocytes among individuals with cardiometabolic risk factors, including obesity, T2DM, dyslipidemia, and hypertension [4]. MASLD should be regarded as a multisystem disorder in which hepatic steatosis, inflammation, and fibrosis are closely linked to metabolic perturbations in adipose tissue, the gut, and peripheral organs [3,13] (Figure 1). This integrated framework provides a biologically coherent explanation of disease heterogeneity and progression.

Hepatic lipid accumulation constitutes an initial permissive condition that increases susceptibility to metabolic and inflammatory injury. Insulin resistance is a central driver of this process, as impaired insulin-mediated suppression of adipose tissue lipolysis leads to increased flux of non-esterified fatty acids to the liver [14,15,16,17]. In parallel, compensatory hyperinsulinemia stimulates de novo lipogenesis through transcriptional regulators such as sterol regulatory element-binding protein 1c and carbohydrate response element-binding protein [17,18,19,20]. When hepatocellular lipid handling capacity is exceeded, bioactive lipid intermediates, including diacylglycerols and ceramides, accumulate [21,22,23]. These lipotoxic species disrupt insulin signaling, impair mitochondrial function, and induce oxidative stress, facilitating hepatocellular ballooning, cell death, and progression from steatosis to MASH [17,24,25].

Persistent lipid overload exerts chronic stress on mitochondrial β-oxidation pathways, resulting in reduced oxidative capacity, impaired ATP production, and excessive generation of reactive oxygen species (ROS) [26,27,28,29]. Oxidative damage to lipids, proteins, and DNA amplifies hepatocellular dysfunction and inflammatory signaling [30,31]. Concurrently, ER stress is triggered by increased demands on lipid synthesis and protein folding. Although the unfolded protein response is initially adaptive, its chronic activation promotes apoptotic and pro-inflammatory signaling cascades, reinforcing disease progression [32,33,34].

Inflammation in MASH is predominantly sterile and initiated by damage-associated molecular patterns released from stressed or dying hepatocytes [35]. These signals activate Kupffer cells and recruit monocyte-derived macrophages, leading to the secretion of pro-inflammatory cytokines such as tumor necrosis factor-α (TNF-α), interleukin-6 (IL-6), and interleukin-1β (IL-1β) [35]. Activation of NLRP3 inflammasomes provides a mechanistic link between metabolic stress and innate immune activation, sustaining inflammatory and fibrogenic feed-forward loops [36]. Chronic inflammatory signaling induces macrophage-derived profibrogenic mediators, such as transforming growth factor-β (TGF-β), which drive hepatic stellate cell (HSC) activation and progressive fibrosis [37]. Fibrosis severity remains the strongest predictor of liver-related morbidity and mortality in MASLD [38]. Extrahepatic organ crosstalk further influences disease progression. Dysfunctional adipose tissue promotes systemic insulin resistance and chronic low-grade inflammation through altered adipokine secretion and immune cell infiltration, forming the adipose–liver axis [23,39,40]. In parallel, the gut–liver axis plays a central role; gut microbiota dysbiosis and increased intestinal permeability permit translocation of microbial products into the portal circulation, thereby activating hepatic innate immune pathways and exacerbating inflammation and fibrogenesis [41].

Collectively, MASLD pathogenesis reflects an interconnected network of lipid accumulation, lipotoxicity, inflammation, and fibrogenesis, which is regulated at transcriptional and epigenetic levels. Among emerging regulatory factors, SIRT6 has been implicated in the modulation of key pathogenic processes, including hepatic lipogenesis, inflammatory signaling, and fibrotic responses. Although the precise mechanisms remain incompletely defined, existing evidence suggests that SIRT6 operates at upstream regulatory nodes integrating metabolic and inflammatory cues. The role of SIRT6 in coordinating these pathogenic pathways will be discussed in detail in the following sections of this review.

## 3. Biological Functions of the Histone Deacetylase SIRT6

### 3.1. Molecular Architecture and Cellular Localization

**Figure 2:**
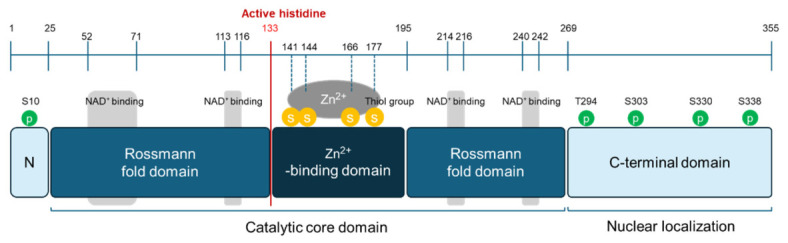
Domain architecture of SIRT6. SIRT6 consists of a conserved catalytic core composed of Rossmann fold domains flanking a Zn^2+^-binding domain, which is essential for structural stability. The enzyme utilizes NAD^+^ as a co-substrate, with the active site located within the central catalytic region. The C-terminal domain contributes to chromatin association and protein–protein interactions, while N-terminal regions may also modulate enzymatic activity.

SIRT6 is an evolutionarily conserved member of the class III histone deacetylase family and requires NAD^+^ as an obligate co-substrate, thereby directly coupling its enzymatic activity to cellular metabolic status [42]. The human *SIRT6* gene is located on chromosome 19p13.3 and encodes a 355–amino acid protein with a molecular mass of approximately 39 kDa [8]. At the subcellular level, SIRT6 is predominantly nuclear and constitutively associated with chromatin, distinguishing it from other sirtuins with broader intracellular distribution [43]. Unlike SIRT1, which undergoes dynamic nucleocytoplasmic shuttling [44], SIRT6 remains tightly bound to chromatin throughout the cell cycle, consistent with its role as a stable chromatin-associated regulator. Structurally, SIRT6 consists of a conserved catalytic core flanked by N- and C-terminal extensions that confer substrate specificity, chromatin affinity, and protein–protein interactions. The catalytic core adopts the canonical sirtuin fold, consisting of a Rossmann-fold NAD^+^-binding domain and a zinc-binding domain stabilized by a structural Zn^2+^ ion [45]. Histidine 133 serves as a critical catalytic residue required for enzymatic activity [46]. The N-terminal region mediates high-affinity chromatin binding, whereas the C-terminal extension contains nuclear localization signals and facilitates interactions with chromatin-associated proteins, including LMNA, MYC, and SMARCA5 [47,48] (Figure 2).

SIRT6 is selectively enriched at specific genomic loci, such as telomeres, centromeres, and promoters of stress-responsive genes, enabling locus-specific regulation of transcription and genome stability [49,50].

### 3.2. Enzymatic Plasticity: A Multitasking Chromatin Enzyme

SIRT6 was initially characterized as a histone deacetylase with relatively weak activity toward free histone peptides; however, subsequent biochemical, structural, and cellular studies have revealed a broader enzymatic repertoire encompassing deacetylation, long-chain deacylation, and mono-ADP-ribosylation [51].

SIRT6 preferentially targets acetylated lysine residues on histone H3, particularly H3K9Ac and H3K56Ac, which are epigenetic marks associated with transcriptional activation and genomic instability [52,53]. Removal of these acetyl groups promotes chromatin compaction, transcriptional repression, and preservation of genome integrity. SIRT6 also deacetylates H3K18Ac at pericentric heterochromatin, a function critical for mitotic fidelity and prevention of cellular senescence [50]. Through these activities, SIRT6 establishes repressive chromatin environments at metabolically and stress-responsive loci.

Accumulating evidence indicates that SIRT6 functions as a highly efficient long-chain deacylase and, in certain contexts, exhibits greater catalytic efficiency for fatty acyl substrates than for acetylated lysines [54]. SIRT6 removes myristoyl, palmitoyl, and other long-chain acyl modifications from lysine residues on non-histone proteins, thereby directly linking lipid metabolism to protein function [55]. A prototypical substrate is TNF-α, in which SIRT6-mediated deacylation regulates intracellular trafficking and secretion, modulating systemic inflammatory responses [55]. More recently, SIRT6 has been reported to remove lactyl and β-hydroxybutyryl modifications, positioning it as a sensor and effector of metabolic state-dependent epigenetic marks [56].

Under conditions of genotoxic or metabolic stress, SIRT6 catalyzes mono-ADP-ribosylation reactions using NAD^+^ as a donor [57]. SIRT6 ribosylates poly(ADP-ribose) polymerase 1 (PARP1) at lysine 521, enhancing PARP1 activity and promoting efficient DNA double-strand break repair [57]. In addition, ribosylation of KAP1 by SIRT6 maintains repression of LINE1 retrotransposons, a function that deteriorates with aging and contributes to genomic instability and inflammation [58].

### 3.3. Master Regulation of Metabolism

SIRT6 functions as a master metabolic rheostat, integrating nutrient availability with transcriptional programs governing glucose, lipid, cholesterol, and mitochondrial metabolism.

#### 3.3.1. Glucose Metabolism

Under physiological nutrient conditions, SIRT6 binds promoters of multiple glycolytic genes, including *HK2*, *PFK1*, and *LDHA*, and deacetylates H3K9, thereby repressing their transcription [59]. SIRT6 functions as a co-repressor of hypoxia-inducible factor 1α (HIF-1α), a central regulator of glycolytic gene expression [60,61]. Loss of SIRT6 enhances HIF-1α activity, leading to increased glucose uptake and glycolytic flux. In parallel, mitochondrial respiration is suppressed, consistent with a Warburg-like metabolic shift. SIRT6-deficient mice develop severe hypoglycemia accompanied by markedly elevated glycolysis, underscoring the role of SIRT6 as a metabolic gatekeeper that shifts glucose utilization from glycolysis toward oxidative metabolism [60].

In the liver, SIRT6 regulates gluconeogenesis through interactions with key transcription factors and coactivators. SIRT6 deacetylates FOXO1 and PGC-1α, thereby modulating the transcription of gluconeogenic genes such as *PEPCK* and *G6PC* [62]. p53 induces SIRT6 expression under specific stress conditions. SIRT6 subsequently deacetylates FOXO1, facilitating its nuclear export and attenuating gluconeogenic gene expression when glucose production needs to be limited. Through coordinated regulation of glycolysis and gluconeogenesis, hepatic SIRT6 fine-tunes glucose output in response to nutrient availability and hormonal signals.

#### 3.3.2. Lipid and Cholesterol Metabolism

SIRT6 suppresses hepatic de novo lipogenesis by inhibiting key lipogenic transcription factors, including SREBP1, ChREBP, and LXR [63]. Direct deacetylation of SREBP1 at lysine 289 and ChREBP at lysine 672 reduces transcriptional activity of these factors and limits lipid accumulation.

In parallel, SIRT6 promotes hepatic β-oxidation through physical interaction with and co-activation of PPARα, thereby increasing expression of fatty acid oxidation (FAO) genes [64]. SIRT6 also regulates lipid metabolic enzymes such as acyl-CoA synthetase long-chain family member 5 (ACSL5) [12] and represses miR-122, both of which favor enhanced FAO and reduced hepatic steatosis [12,65].

Beyond fatty acid metabolism, SIRT6 modulates cholesterol homeostasis through epigenetic mechanisms. SIRT6 cooperates with FOXO3 to deacetylate histone H3K9 and H3K56 at the SREBP2 promoter, leading to suppression of SREBP2 and downstream targets, including HMG-CoA reductase [66]. Consistently, liver-specific SIRT6 knockout mice exhibit elevated hepatic and circulating cholesterol levels [66]. In addition, SIRT6 reduces low-density lipoprotein (LDL)-cholesterol by repressing PCSK9 transcription via FOXO3-dependent chromatin recruitment and H3K9/H3K56 deacetylation. This repression increases LDL receptor availability and enhances LDL clearance [67].

#### 3.3.3. Mitochondrial Function

SIRT6 plays a pivotal role in mitochondrial biogenesis and quality control by activating peroxisome proliferator-activated receptor gamma coactivator 1α (PGC-1α) and regulating mitochondrial transcription factor A (TFAM), both of which are essential for mitochondrial DNA replication and transcription [68,69,70]. By preserving mitochondrial integrity and oxidative capacity, SIRT6 reduces oxidative stress and metabolic exhaustion, both of which are central to fatty liver disease progression and age-related metabolic decline [71].

These findings establish SIRT6 as a chromatin-bound, metabolically responsive regulator that integrates epigenetic control with inflammatory signaling and mitochondrial function. Lipid accumulation, insulin resistance, sterile inflammation, oxidative stress, and fibrogenic signaling constitute core pathogenic axes of MASLD. The convergence of SIRT6-regulated pathways on hepatocellular metabolism and stress responses provides a mechanistic rationale for evaluating how SIRT6 dysfunction contributes to MASLD initiation and progression. The following section therefore focuses on the role of SIRT6 as a molecular node linking metabolic dysregulation to hepatic inflammation, fibrosis, and disease severity in MASLD.

## 4. Role of SIRT6 in MASLD Pathogenesis

### 4.1. Suppression of Hepatic Lipogenesis by SIRT6

**Figure 3:**
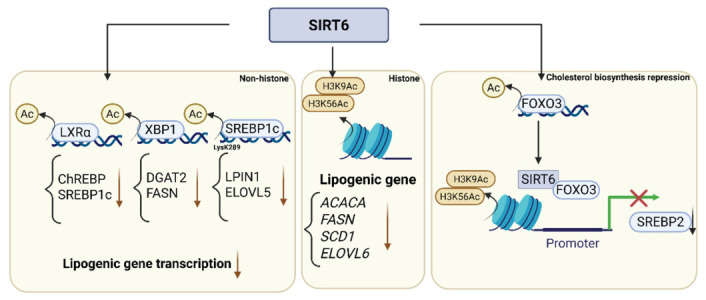
SIRT6 represses hepatic lipogenesis via epigenetic and transcriptional mechanisms. SIRT6 suppresses lipogenic gene expression through coordinated histone and non-histone deacetylation. SIRT6 removes H3K9Ac and H3K56Ac at the promoters of lipogenic genes (*ACACA*, *FASN*, *SCD1*, and *ELOVL6*), leading to transcriptional repression. In parallel, SIRT6 deacetylates LXRα, XBP1, and SREBP1c, thereby inhibiting downstream lipogenic programs. SIRT6 suppresses cholesterol biosynthesis via FOXO3-dependent inhibition of SREBP2. Created in BioRender. Hwang, S. (2026). BioRender.com/pt90osz (accessed on 7 March 2026).

SIRT6 functions as an epigenetic regulator of hepatic de novo lipogenesis, a major source of triglyceride accumulation in MASLD [63]. SIRT6 interacts with and deacetylates lipogenic transcription factors, including LXRα, ChREBP, SREBP1c, and XBP1, thereby repressing fatty acid and cholesterol biosynthesis [63,72,73] (Figure 3).

SIRT6 regulates lipogenic gene expression through histone and non-histone deacetylation. Deacetylation of histone H3K9 and H3K56 reduces transcription of genes encoding lipogenic enzymes such as ACACA, FASN, SCD1, ELOVL6, and DGAT2 [63]. In addition, SIRT6 deacetylates specific lysine residues on transcription factors to modulate transcriptional activity [63,74]. For example, deacetylation of SREBP1c at Lys289 regulates the expression of LPIN1 and ELOVL5 [63].

LXRα regulates transcription of both ChREBP and SREBP1c [75,76]. Therefore, inhibition of LXRα by SIRT6 suppresses lipogenesis through both direct and indirect mechanisms. SIRT6 activity attenuates nutrient- and hormone-induced lipogenic signaling, including glucose-dependent activation of ChREBP and insulin-mediated activation of SREBP1c.

SIRT6 also regulates circadian control of hepatic lipogenesis. SIRT6 interacts with CLOCK and BMAL1 and deacetylates H3K9 at lipogenic gene promoters, thereby limiting transcriptional activation to appropriate circadian phases [77]. Hepatocyte-specific deletion of SIRT6 disrupts temporal regulation, resulting in persistent lipogenic gene expression under basal and high-nutrient conditions [77].

In addition to triglyceride metabolism, SIRT6 regulates cholesterol biosynthesis through repression of SREBP2 [66]. SIRT6 deacetylates FOXO3, promoting its recruitment to the SREBP2 promoter and facilitating deacetylation of H3K9 and H3K56, resulting in transcriptional repression [66]. SIRT6 knockout mice develop hepatic steatosis and hypercholesterolemia [66,72]. In human MASLD, reduced hepatic SIRT6 expression is inversely correlated with lipogenic gene expression. Restoration of SIRT6 expression via adeno-associated virus-mediated overexpression reduces steatosis and lipogenic gene expression in multiple experimental models [63,66].

### 4.2. Regulation of Fatty Acid Oxidation and Mitochondrial Homeostasis by SIRT6

**Figure 4:**
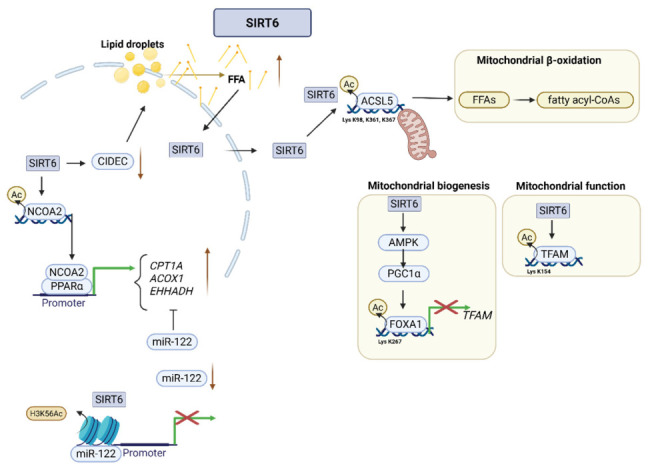
SIRT6 regulates fatty acid oxidation and mitochondrial function. SIRT6 enhances lipid catabolism by activating the NCOA2/PPARα axis and inducing β-oxidation genes (*CPT1A*, *ACOX1*, and *EHHADH*), while repressing miR-122. SIRT6 deacetylates ACSL5, facilitating fatty acyl-CoA formation and promoting mitochondrial β-oxidation. In addition, SIRT6 regulates mitochondrial biogenesis and quality control via AMPK/PGC-1α signaling, with mitochondrial transcription factor A (TFAM) serving as a central regulatory node. By deacetylating FOXA1 to repress TFAM expression and directly deacetylating TFAM, SIRT6 regulates mitochondrial DNA transcription and replication, thereby limiting basal mitochondrial biogenesis while preserving stress-induced adaptive expansion. Created in BioRender. Hwang, S. (2026). BioRender.com/pt90osz (accessed on 7 March 2026).

SIRT6 promotes hepatic FAO and mitochondrial homeostasis through a coordinated network of transcriptional, epigenetic, and post-translational mechanisms [64] (Figure 4). Inside the nucleus, SIRT6 acts as a critical coactivator for peroxisome proliferator-activated receptor alpha (PPARα). Deacetylation of nuclear receptor coactivator 2 (NCOA2) at lysine 780 enhances recruitment of NCOA2 to PPARα target promoters and increases expression of FAO-related enzymes, such as CPT1A, ACOX1, and EHHADH [64]. SIRT6 also represses microRNA-122 (miR-122) through deacetylation of histone H3K56 at the miR-122 promoter [65]. Because miR-122 inhibits FAO-associated genes, repression of miR-122 enhances oxidative metabolism in the liver [65].

SIRT6 directly regulates lipid substrate mobilization and activation [78]. SIRT6 suppresses the expression of cell death-inducing DFFA-like effector C (CIDEC), a lipid droplet protein-associated protein that inhibits lipolysis [78]. A reduction in CIDEC increases the release of free fatty acids from lipid droplets. Subsequently, long-chain fatty acids such as palmitate trigger SIRT6 translocation to the cytoplasm, where SIRT6 deacetylates ACSL5 at lysine residues K98, K361, and K367 [12]. Deacetylation of ACSL5 increases the conversion of free fatty acids into fatty acyl-CoAs for mitochondrial β-oxidation [12]. The clinical relevance of this axis is underscored by findings in patients with MASH, who exhibit reduced SIRT6 and hyperacetylated (inactive) ACSL5, correlating with impaired lipid catabolism [12].

SIRT6 also governs mitochondrial biogenesis and quality through pathways involving PGC-1α and AMPK [70]. A central node in this regulation is TFAM [70]. SIRT6 deacetylates FOXA1 at lysine 267 to repress TFAM gene expression and directly deacetylates TFAM protein at lysine 154 [70]. These mechanisms regulate mitochondrial DNA transcription and replication. This prevents excessive basal mitochondrial biogenesis while allowing expansion during energetic stress. Furthermore, SIRT6 maintains the expression of mitochondrial sirtuins SIRT3 and SIRT4, which are required for oxidative phosphorylation and ROS detoxification.

The physiological consequence of SIRT6 deficiency is a profound metabolic shift [72]. Hepatocyte-specific deletion of SIRT6 impairs FAO and enhances reliance on glycolysis and de novo lipogenesis, resulting in steatosis and accumulation of glycolytic metabolites such as pyruvate [72]. Conversely, SIRT6 overexpression induces pyruvate dehydrogenase kinase 4 (PDK4) to suppress glucose oxidation, prioritizing fatty acid utilization [64]. These findings establish SIRT6 as a master regulator that integrates nuclear transcription, cytoplasmic enzyme activation, and mitochondrial quality control to sustain oxidative metabolism and prevent pathological lipid storage [12,64].

### 4.3. Regulation of Very Low-Density Lipoprotein Secretion and Bile Acid Homeostasis by SIRT6

The export of lipids via very low-density lipoprotein (VLDL) particles plays an important role in governing hepatic lipid secretion and systemic lipid distribution [79]. SIRT6 regulates this process through mechanisms linked to bile acid homeostasis. At the molecular level, SIRT6 inhibits hepatic bile acid synthesis by targeting estrogen-related receptor gamma (ERRγ), a transcriptional activator of cholesterol 7α-hydroxylase (CYP7A1), the rate-limiting enzyme in bile acid production [80,81,82]. SIRT6 deacetylates ERRγ and promotes proteasomal degradation, thereby reducing ERRγ protein stability and transcriptional activity [81]. Decreased ERRγ activity leads to reduced CYP7A1 expression, which limits hepatic conversion of cholesterol to bile acids and decreases the overall bile acid pool [81,82,83]. Through this mechanism, SIRT6 modulates cholesterol flux into the bile acid synthesis pathway and influences downstream processes that regulate hepatic lipid handling and VLDL secretion [81,84].

The functional consequences of SIRT6-dependent regulation of bile acid synthesis are mediated, in part, through intestinal lipid absorption. Adeno-associated virus (AAV)-mediated SIRT6 overexpression reduces hepatic CYP7A1 expression and decreases the bile acid pool [84]. Reduced bile acid availability modulates signaling pathways involving fibroblast growth factor 15/19 (FGF15/19) and the farnesoid X receptor (FXR), leading to decreased intestinal absorption of cholesterol and fatty acids [85,86,87]. Reduced intestinal lipid uptake limits delivery of lipid substrates to the liver and results in decreased secretion of VLDL-cholesterol and VLDL-triglyceride [84,88].

In contrast, hepatocyte-specific SIRT6 deletion increases CYP7A1 expression, expands the bile acid pool, and enhances intestinal lipid absorption, thereby increasing substrate availability for hepatic VLDL production [84]. Regulation of VLDL secretion by SIRT6 is substrate-dependent. Modulation of SIRT6 does not alter the expression of structural apolipoproteins, including APOB and APOE. These findings indicate that SIRT6 regulates VLDL output by controlling bile acid-dependent lipid substrate availability rather than by directly affecting the VLDL assembly process.

### 4.4. Inflammation and Endoplasmic Reticulum Stress

Hepatic inflammation and ER stress are central drivers of disease progression from simple steatosis to MASH [89,90]. These processes are driven by disrupted lipid homeostasis and metabolic overload, which activate both cell-intrinsic stress responses and extrinsic immune-mediated inflammatory pathways [91,92]. SIRT6 attenuates these pathogenic processes through distinct yet complementary mechanisms operating in hepatocytes and immune cells.

**Figure 5:**
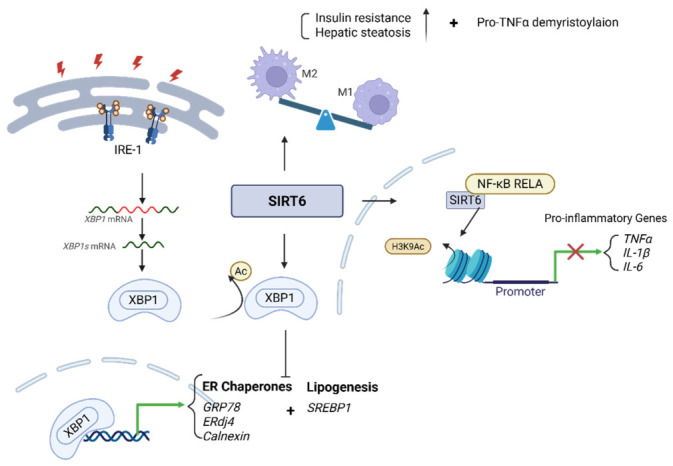
SIRT6 attenuates endoplasmic reticulum (ER) stress and inflammation. SIRT6 limits ER stress by suppressing the IRE1–XBP1 signaling pathway through XBP1 deacetylation, thereby reducing the expression of ER chaperones (*GRP78*, *ERdj4*, *Calnexin*) and lipogenic signaling. Concurrently, SIRT6 represses inflammatory responses via NF-κB (RELA) deacetylation, leading to decreased expression of pro-inflammatory cytokines (*TNF-α*, *IL-1β*, and *IL-6*). Created in BioRender. Hwang, S. (2026). BioRender.com/pt90osz (accessed on 7 March 2026).

In the context of ER stress, SIRT6 modulates signaling through the inositol-requiring enzyme 1α (IRE1α)–X-box binding protein 1 (XBP1) axis, a major branch of the unfolded protein response (UPR) [74,93] (Figure 5). Under conditions of ER stress induced by unfolded protein accumulation, lipid saturation, or calcium imbalance, IRE1α undergoes autophosphorylation and acquires endoribonuclease activity, catalyzing the unconventional splicing of XBP1 mRNA to generate the active transcription factor XBP1s [94]. Nuclear XBP1s induce expression of ER chaperones, including GRP78, ERdj4, and calnexin, while also activating lipogenic transcriptional programs involving SREBP1 and enzymes required for triglyceride synthesis [95,96]. In MASLD, this lipogenic component of the UPR represents a maladaptive response that enhances hepatic lipid accumulation and perpetuates ER stress, thereby establishing a self-reinforcing pathogenic loop [97,98].

SIRT6 directly deacetylates XBP1s, thereby limiting its transcriptional activity and attenuating both ER stress-associated lipogenic gene induction and downstream metabolic dysfunction [74]. Hepatocytes lacking SIRT6 exhibit exaggerated responses to tunicamycin-induced ER stress, including marked activation of XBP1s signaling, upregulation of the protein kinase R-like ER kinase (PERK) pathway, increased expression of ER stress markers such as GRP78 and activating transcription factor 6 (ATF6), and excessive accumulation of intracellular triglycerides. In contrast, viral-mediated SIRT6 overexpression abolishes tunicamycin-induced lipid accumulation and suppresses ER stress marker expression in hepatocytes, demonstrating that SIRT6 confers robust protection against ER stress-driven hepatic steatosis.

Beyond hepatocyte-intrinsic effects, SIRT6 suppresses hepatic inflammation through epigenetic regulation in both parenchymal and immune cells [8,99]. SIRT6 interacts with the NF-κB RELA subunit and deacetylates histone H3K9 at promoters of pro-inflammatory genes, thereby reducing chromatin accessibility and transcription of cytokines such as TNF-α, IL-1β, and IL-6 [52]. Loss of SIRT6 results in increased histone acetylation at these loci and enhanced NF-κB-dependent gene expression, contributing to inflammatory amplification during disease progression [100,101].

In macrophages, SIRT6 regulates phenotypic polarization [102,103]. Myeloid-specific SIRT6 promotes a shift toward the pro-inflammatory M1 phenotype and exacerbates insulin resistance and hepatic steatosis [102]. In addition, SIRT6 exerts post-translational regulation in the cytoplasm by catalyzing demyristoylation of pro-TNF-α, a modification that reduces cytokine secretion [55,104]. Through these nuclear and cytoplasmic mechanisms, SIRT6 attenuates immune-mediated liver injury and mitigates inflammatory progression in MASH.

SIRT6 regulates macrophage polarization in a context-dependent manner. In acute injury, it promotes M2 polarization, enhancing autophagy and tissue repair while limiting excessive inflammation [105,106,107]. However, prolonged M2 activation under chronic conditions can drive fibrosis through sustained production of TGF-β and other profibrotic mediators, indicating a temporal dual role in inflammation resolution versus fibrotic progression [108,109,110].

Its function is highly sensitive to metabolic and environmental cues. Under hyperglycemia, disrupted NAD+ metabolism reduces SIRT6 activity, impairing efferocytosis and delaying inflammation resolution [111]. In contrast, during Mycobacterium tuberculosis infection, SIRT6 suppresses HIF1-α-dependent glycolysis, weakening antimicrobial responses [112]. In PM2.5-induced inflammation, SIRT6 can instead promote cytokine production and airway inflammation via autophagy [113]. These findings highlight that SIRT6 acts as a dynamic regulator rather than a uniformly anti-inflammatory factor.

Beyond macrophages, SIRT6 also modulates other immune cells. In ILC3, SIRT6 represses IL-22 production through inhibition of HIF1-α and mitochondrial ROS, thereby limiting mucosal immune responses [114]. In adaptive immunity, SIRT6 regulates dendritic cell activation and T cell differentiation, promoting regulatory T cells while suppressing Th1/Th17 responses [115,116]. In tumor environments, SIRT6 contributes to NK cell exhaustion, whereas its inhibition restores cytotoxic function [117].

Overall, SIRT6 functions as a metabolic epigenetic checkpoint that dynamically regulates immune responses across cell types. Its effects vary depending on metabolic state, pathogen context, and stage of inflammation. Importantly, the vast majority of studies investigating the immunoregulatory roles of SIRT6 have been conducted in extrahepatic tissues, highlighting a critical gap in our understanding of its role within the liver. Therefore, further studies focusing on SIRT6 in hepatic immune contexts are essential to fully elucidate its immunological and pathological significance.

### 4.5. Regulation of Hepatic Fibrosis by SIRT6

**Figure 6:**
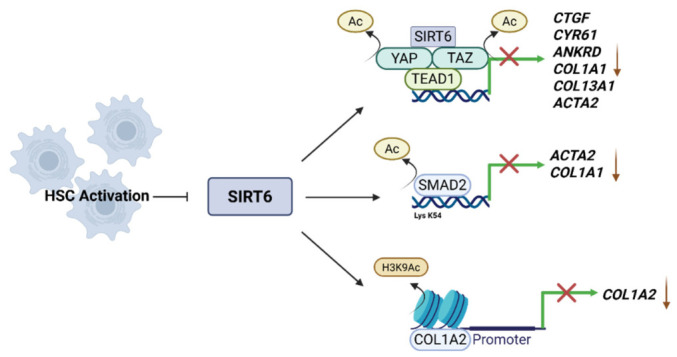
SIRT6 inhibits hepatic stellate cell (HSC) activation and fibrogenesis. SIRT6 suppresses fibrogenesis by deacetylating YAP/TAZ and SMAD2, thereby inhibiting the expression of pro-fibrotic genes (*CTGF*, *CYR61*, *ANKRD1*, *COL1A1*, *COL13A1*, and *ACTA2*). SIRT6 further represses *COL1A2* transcription via histone deacetylation. Collectively, these mechanisms limit HSC activation and extracellular matrix deposition. Created in BioRender. Hwang, S. (2026). BioRender.com/pt90osz (accessed on 7 March 2026).

SIRT6 functions as a critical negative regulator of HSC activation and fibrogenesis through coordinated suppression of multiple convergent pro-fibrotic signaling pathways [11,118] (Figure 6). A principal pathway regulated by SIRT6 is the TGF-β–SMAD axis. SIRT6 directly interacts with and deacetylates SMAD2 and SMAD3, thereby limiting their nuclear translocation and transcriptional activity [11,118]. Mass spectrometry analysis has identified lysine 54 (K54) of SMAD2 as a key deacetylation site. Substitution of this residue (K54R) abolishes SIRT6-dependent regulation, indicating that this specific post-translational modification is essential for the suppression of fibrogenic signaling.

In activated HSCs and fibrotic liver tissue, SIRT6 expression is progressively reduced. This reduction facilitates SMAD hyperacetylation and sustains transcription of fibrogenic genes [118]. In addition to the TGF-β–SMAD pathway, SIRT6 suppresses the activity of the Hippo pathway coactivators YAP and TAZ, which promote myofibroblast transdifferentiation and extracellular matrix production [119,120,121]. Accordingly, HSC-specific deletion of SIRT6 exacerbates diet-induced hepatic fibrosis and is associated with enhanced TGF-β–SMAD3 and Hippo–YAP/TAZ signaling.

Restoration of SIRT6 activity, either through genetic overexpression or pharmacological activation using the selective agonist MDL-800, significantly attenuates fibrogenesis [118]. MDL-800 has been reported to decrease SMAD2 acetylation and downregulate key profibrotic markers, including α-smooth muscle actin (α-SMA) and collagen type I alpha 1 (COL1A1). Beyond post-translational regulation of SMAD proteins, SIRT6 exerts epigenetic control by deacetylating histone H3K9 at fibrogenic promoters, such as COL1A2, thereby decreasing chromatin accessibility and transcriptional output [11]. Notably, TGF-β stimulation can transiently induce SIRT6 expression through an SMAD3- and SPTBN1-dependent mechanism, establishing a negative feedback loop that limits excessive profibrotic signaling [122].

Collectively, these post-translational and epigenetic mechanisms position SIRT6 as a central regulatory node restraining HSC activation and fibrosis progression. Therefore, modulation of SIRT6 activity represents a mechanistically rational therapeutic strategy to limit fibrotic progression and potentially reverse pathological remodeling in advanced MASLD.

### 4.6. Regulation of Adipose–Liver Axis by SIRT6

The adipose–liver axis represents a critical component in the pathogenesis of MASLD, particularly under conditions of insulin resistance. In dysfunctional adipose tissue, enhanced lipolysis leads to excessive release of free fatty acids, which are subsequently taken up by hepatocytes, promoting lipid accumulation and lipotoxicity. Therefore, regulatory mechanisms within adipose tissue can profoundly influence hepatic metabolic homeostasis. SIRT6 has emerged as an important modulator of adipose tissue function. Adipocyte-specific deletion of *Sirt6* in mice results in increased body weight and adipose tissue mass, accompanied by impaired lipolytic capacity [78]. Consistently, Xiong et al. demonstrated that Sirt6 ablation using *Fabp4*-Cre mice leads to exacerbated adiposity, glucose intolerance, and systemic insulin resistance [123]. Furthermore, *Sirt6* deficiency in adipose tissue is associated with upregulation of pro-inflammatory gene expression in white adipose depots, indicating a shift toward a metabolically unfavorable and inflammatory state. Collectively, these findings suggest that SIRT6 plays a protective role in maintaining adipose tissue metabolic integrity by coordinating lipolysis and suppressing inflammation, thereby preserving systemic insulin sensitivity. Through these mechanisms, adipose SIRT6 may indirectly attenuate hepatic steatosis and inflammation, ultimately influencing the progression of MASLD.

## 5. Therapeutic Strategies Targeting SIRT6 Activation in MASLD

The expanding recognition of SIRT6 as a central regulator of hepatic lipid metabolism, inflammation, and fibrogenesis has positioned pharmacological activation of SIRT6 as a rational therapeutic strategy for MASLD. Advances in SIRT6-targeted pharmacology have yielded several therapeutic modalities, including selective small-molecule activators, bioactive natural compounds, and gene delivery approaches, each demonstrating efficacy in preclinical models by ameliorating hepatic steatosis, suppressing inflammatory signaling, and limiting fibrotic progression.

### 5.1. Selective Small-Molecule SIRT6 Activators

The development of potent and selective small-molecule SIRT6 activators represents a major pharmacological advance [124,125,126,127,128] (Table 1). UBCS039, a well-characterized SIRT6-selective activator, has demonstrated anti-steatotic and anti-inflammatory effects in both cellular and animal models of MASLD through direct enhancement of SIRT6 deacetylase activity [44,129]. In hepatocyte culture models, UBCS039 suppressed LXRα expression and transcriptional activity induced by LXR agonists via SIRT6-dependent deacetylation of LXRα at its principal lysine residues. This reduction in LXRα acetylation provides direct biochemical evidence of SIRT6 activation and results in downstream repression of SREBP1c expression and activity. Consequently, expression of SREBP1c-regulated lipogenic genes, including *ACACA*, *FASN*, *SCD1*, and *DGAT2*, was markedly reduced. Importantly, genetic knockdown of SIRT6 abolished these effects, confirming the SIRT6-dependence of UBCS039-mediated lipogenic suppression [44].

Functionally, UBCS039 attenuated lipid accumulation in hepatocytes exposed to LXR agonists or saturated fatty acids, such as palmitic acid, establishing the anti-steatotic effect of UBCS039 [44]. In addition, UBCS039 exerted anti-inflammatory effects by suppressing NF-κB p65 expression and pro-inflammatory cytokine production in hepatocytes subjected to combined lipotoxic and oxidative stress conditions that recapitulate key pathogenic features of MASLD [129]. In vivo, UBCS039 administration attenuated LXR agonist-induced hepatic steatosis in mice, supporting its translational potential [44].

### 5.2. Structure-Based Design of Dual LXR/SIRT6-Targeting Compounds

Structure-guided medicinal chemistry efforts have identified squaramide-based compounds that simultaneously inhibit the LXR/SREBP-1c lipogenic pathway and activate SIRT6, thereby achieving dual metabolic benefit within a single molecular framework. Through rational structure-activity relationship optimization, a series of *N*-aryl-*N*’-[4-(aryloxy)cyclohexyl] squaramides was generated, with compound 31 emerging as a lead candidate. Compound 31 potently suppressed LXRα and SREBP1c expression and activity in hepatocyte-based reporter assays, with mechanistic studies indicating that its anti-lipogenic effects were mediated, at least in part, through upstream activation of SIRT6. In HepG2 cells, compound 31 reduced intracellular triglyceride and cholesterol accumulation compared with vehicle-treated controls [130].

In vivo efficacy of compound 31 has been demonstrated in high-fat diet-fed mice through attenuation of hepatic lipid accumulation and suppression of the expression of lipogenic genes, including *ACACA*, *FASN*, and *SCD1* [130]. Although compound 31 indirectly activated SIRT6 in biochemical assays, its inhibitory effects on the LXR/SREBP-1c pathway were reversed by SIRT6 knockdown or pharmacologic SIRT6 inhibition, indicating SIRT6-dependent modulation of hepatic lipogenesis. These findings underscore the therapeutic potential of structure-based strategies that integrate SIRT6 activation with targeted suppression of lipogenic transcriptional programs.

### 5.3. Natural Products as SIRT6 Activators

Several naturally occurring compounds have been identified as functional SIRT6 activators with therapeutic relevance to MASLD. Atractylenolide I (ATL I), a sesquiterpene lactone isolated from *Atractylodes macrocephala* Koidz, has emerged as a highly promising therapeutic candidate [131]. Molecular docking and biochemical analyses demonstrated direct binding of ATL I to SIRT6 and activation of its deacetylase activity. In hepatocytes, ATL I enhanced SIRT6 activity, leading to activation of PPARα signaling and suppression of SREBP1c-dependent lipogenic gene expression. In diet-induced obesity mouse models, ATL I administration reduced hepatic steatosis, attenuated hepatic inflammatory cytokine production (particularly IL-1β and TNF-α), and inhibited NLRP3 inflammasome activation through SIRT6-dependent mechanisms. Notably, these protective effects were diminished in hepatocyte-specific SIRT6-deficient mice, establishing SIRT6 as the primary mediator of ATL I activity [131].

Additional natural compounds, including ginsenosides, have also been shown to exert SIRT6-dependent metabolic benefits. Ginsenoside Rd activated SIRT6 in hepatocytes, leading to enhanced PPARα-mediated fatty acid β-oxidation and reductions in lipid accumulation, oxidative stress, and inflammatory signaling in vitro and in vivo [132]. Similarly, ginsenoside Rc protected against high-fat diet-induced hepatic steatosis and inflammation through SIRT6-dependent activation of PPARα and FAO pathways [133].

### 5.4. Gene Delivery for SIRT6 Restoration

Gene therapy approaches aimed at restoring hepatic SIRT6 expression have demonstrated efficacy in preclinical MASLD models. AAV-mediated hepatic overexpression of SIRT6 ameliorated Western diet-induced hepatic steatosis and atherosclerosis in LDL receptor-deficient mice [63,84]. SIRT6 overexpression reduced hepatic triglyceride and cholesterol contents, suppressed lipogenic transcriptional programs mediated by LXRα, ChREBP, and SREBP1c, and enhanced PPARα-driven FAO. In parallel, AAV-SIRT6 improved mitochondrial biogenesis through activation of the PGC-1α-TFAM axis and attenuated hepatic inflammation by suppressing NF-κB-dependent inflammatory signaling. Importantly, SIRT6 restoration also limited fibrotic progression in this model, underscoring its broad therapeutic impact across multiple stages of MASLD pathogenesis [84].

Although AAV-based gene delivery offers sustained therapeutic expression following a single administration, its clinical translation is constrained by regulatory, immunogenicity, manufacturing, and scalability challenges. These considerations necessitate cautious evaluation prior to widespread clinical implementation [134].

## 6. Conclusions and Future Perspectives

Despite major advances in defining the molecular basis of MASLD, current therapeutic strategies remain largely pathway-specific and do not sufficiently address the integrated regulatory networks that drive disease initiation, progression, and heterogeneity. The evidence reviewed herein identifies SIRT6 as a central epigenetic and metabolic regulator positioned at the convergence of these pathogenic processes.

SIRT6 regulates hepatic physiology through coordinated control of chromatin accessibility, transcriptional programs, mitochondrial function, and inflammatory signaling. By integrating metabolic, inflammatory, and fibrogenic pathways, SIRT6 functions as a critical molecular constraint on MASLD progression. Its enzymatic activity is tightly coupled to intracellular NAD^+^ availability, directly linking SIRT6-dependent regulatory capacity to cellular metabolic state. Consistent reductions in hepatic SIRT6 expression and activity across experimental models and human MASLD cohorts underscore its pathophysiological relevance.

From a therapeutic standpoint, targeting SIRT6 offers conceptual advantages over interventions that modulate isolated downstream pathways. Activation of SIRT6 has the potential to simultaneously ameliorate hepatic lipid accumulation, metabolic inflexibility, inflammatory signaling, and fibrogenesis. Preclinical studies employing small-molecule activators, natural compounds, and gene-based approaches provide proof of principle that restoration of SIRT6 activity can attenuate steatosis, suppress inflammatory responses, and limit fibrosis progression. Collectively, these findings support the notion that SIRT6-directed strategies may exert disease-modifying effects rather than providing symptomatic benefit alone.

Several challenges remain to be addressed. The context-dependent functions of SIRT6 across distinct hepatic cell populations require careful evaluation, particularly with respect to sustained activation and long-term safety. In addition, the development of selective, bioavailable, and clinically tractable SIRT6 activators remains a key translational priority. Identification of biomarkers that reflect hepatic SIRT6 activity and enable patient stratification will also be essential for clinical translation.

Despite the promising preclinical efficacy of SIRT6-targeted therapies, several critical challenges must be addressed prior to successful translation into human clinical trials. For small-molecule activators and natural compounds, achieving adequate in vivo bioavailability and high target selectivity remains essential to ensure clinical applicability while minimizing off-target toxicity. Moreover, the context-dependent roles of SIRT6 across diverse hepatic and immune cell populations necessitate comprehensive evaluation of the long-term safety associated with sustained systemic activation.

In the context of gene therapy, although AAV-mediated SIRT6 delivery has demonstrated robust and durable therapeutic effects in murine models, its clinical translation remains limited by host immunogenicity, manufacturing constraints, and complex regulatory requirements. Finally, there is a lack of validated, non-invasive biomarkers that reliably reflect hepatic SIRT6 target engagement in humans. The identification and validation of such biomarkers will be crucial for patient stratification and for accurately assessing therapeutic efficacy in future clinical studies.

In conclusion, SIRT6 represents a unifying molecular node integrating metabolic and epigenetic regulation with inflammatory and fibrotic signaling in MASLD. Continued mechanistic and translational investigation of SIRT6-targeted strategies may open new avenues for addressing unmet therapeutic needs associated with MASLD and its progressive complications.

## Figures and Tables

**Table 1:** Small-molecule compounds with MASLD-attenuating effects.

Compound	Model	Effect	Reference
UBCS039	- AML12, HepG2, Huh7 - Lipogenic gene induction by LXR agonist treatment	Reduced lipogenic gene expression (ACACA, FASN, SCD1, DGAT2); suppressed de novo lipogenesis and triglyceride accumulation via modulation of SREBP1c pathway	[44]
	- HepG2, primary hepatocytes - Inflammation induced by LPS, PA, and tBHP	Decreased expression of pro-inflammatory cytokines (IL-1β, IL-6, TNF-α); attenuated ROS production and NF-κB activation	[44]
	- C57BL/6 mice - LXR agonist-induced hepatic steatosis	Alleviated hepatic lipid accumulation and inflammation; reduced hepatic TG content and lipogenic gene expression	[44]
Compound 31	- HepG2, primary hepatocytes - LXR agonist-induced steatosis	Decreased intracellular lipid droplet accumulation; downregulated lipogenesis-related genes (ACACA, FASN, SCD1)	[130]
	- C57BL/6 mice - High-fat diet (HFD)-induced steatosis	Improved hepatic steatosis and lipid metabolic profile; decreased expression of lipogenic genes (ACACA, FASN, SCD1) and reduced hepatic TG content	[130]
Atractylenolide I	- HepG2, primary hepatocytes - Oleic acid/palmitic acid-induced lipid accumulation	Suppressed SREBP1c-dependent lipogenesis, leading to reduced intracellular triglyceride synthesis	[131]
	- C57BL/6 mice - High-fat diet (HFD)-induced steatosis	Decreased hepatic steatosis and inflammatory cytokine expression (IL-1β, TNF-α); improved hepatic histology and serum lipid profile	[131]
Ginsenoside Rd	- Primary hepatocytes - Oleic acid/palmitic acid-induced lipid accumulation	Reduced lipid accumulation and oxidative stress; improved antioxidant enzyme (SOD, CAT) activity and reduced inflammatory cytokines	[132]
	- C57BL/6 mice - High-fat diet (HFD)-induced steatosis	Attenuated hepatic steatosis and inflammation; reduced lipid peroxidation and improved antioxidant balance	[132]
Ginsenoside Rc	- Primary hepatocytes - Oleic acid/palmitic acid-induced lipid accumulation	Inhibited OA/PA-induced lipid deposition; enhanced PPAR-α-mediated fatty acid oxidation	[133]
	- C57BL/6 - High-fat diet (HFD)-induced hepatic steatosis	Decreased hepatic lipid accumulation and inflammatory cytokine levels (TNF-α, IL-6); improved hepatic architecture and metabolic parameters	[133]

## Data Availability

No new data were created or analyzed in this study. Data sharing is not applicable to this article.
